# Low CD4 count and educational status predict abnormal cervical smears amongst HIV-positive women initiating antiretroviral therapy in South Africa

**DOI:** 10.4102/sajhivmed.v21i1.1045

**Published:** 2020-03-30

**Authors:** Nondumiso Mthembu, Jienchi Dorward, Nivashnee Naicker, Farzana Osman, Siphesihle Gumede, Yukteshwar Sookrajh, Paul Drain, Nigel Garrett

**Affiliations:** 1 Centre for the AIDS Programme of Research in South Africa (CAPRISA), University of KwaZulu-Natal, Durban, South Africa; 2 Nuffield Department of Primary Care Health Sciences, University of Oxford, Oxford, United Kingdom; 3 Prince Cyril Zulu Communicable Disease Centre, eThekwini Municipality, Durban, South Africa; 4 Department of Global Health, Schools of Medicine and Public Health, University of Washington, Seattle, United States; 5 Department of Medicine, School of Medicine, University of Washington, Seattle, United States; 6 Department of Epidemiology, School of Public Health, University of Washington, Seattle, United States; 7 Department of Public Health Medicine, School of Nursing and Public Health, University of KwaZulu-Natal, Durban, South Africa

## Introduction

Cervical cancer is common amongst human immunodeficiency virus (HIV)-positive women in low- and middle-income countries. In South Africa, more than 7500 cases are diagnosed annually and over 50% result in death, making cervical cancer the leading cause of cancer mortality.^1^ South Africa’s high HIV prevalence contributes to this high burden, because HIV-positive women are more likely to have persistent human papilloma virus (HPV) infection and precancerous cervical changes.^2^ Cervical cancer is preventable either through HPV vaccination of girls before sexual debut, which was rolled out in South Africa from 2014, or screening and treatment of precancerous cervical lesions. South African guidelines recommend cervical screening for all HIV-positive women at HIV diagnosis and then every 3 years.^1^

Antiretroviral therapy (ART) causes immune reconstitution and may reduce the risk of cervical cancer amongst HIV-positive women by lowering HPV acquisition, increasing HPV clearance and slowing the progression to precancerous lesions.^2^ However, these effects may be diminished for women who initiate ART at low CD4 counts.^3^ Since 2016, when universal test and treat (UTT) was introduced in South Africa, women began initiating ART at CD4 counts > 500 cells/mm^3^ (early initiators) and may therefore be protected against precancerous cervical abnormalities and cancer.^3^

In this study, we aimed to assess whether early initiators of ART had a lower risk of abnormal cervical smears when compared to late initiators (women with a CD4 ≤ 500 cells/mm^3^), after introduction of UTT in South Africa.

## Methods

### Study design

We performed a cross-sectional analysis at enrolment into the Simplifying HIV TREAtment and Monitoring (STREAM) study, a randomised trial assessing point-of-care viral load testing amongst people living with HIV receiving ART (NCT03066128).^4,5^

### Participants and setting

The STREAM study was conducted at the Prince Cyril Zulu Communicable Disease Centre (PCZ CDC), a large public clinic in central Durban, South Africa. At PCZ CDC, all women who test HIV-positive are referred for ART initiation and have CD4 cell count and Papanicolaou cervical smear testing performed using routine National Health Laboratory Services (NHLS). We enrolled non-pregnant, HIV-positive adults aged 18 years or older who were clinically stable on first-line ART for 6 months, and randomised them to receive point-of-care viral load monitoring and task-shifting to an enrolled nurse or standard laboratory monitoring and professional nurse care.^4^ All participants had been initiated on ART after the introduction of UTT. Cervical smear results were checked using NHLS records for all women at screening. Women with abnormal cervical smear results of high-grade squamous intraepithelial lesion (HSIL) who needed referral for colposcopy did not meet the STREAM study eligibility criteria of being clinically stable (as they required active care by a physician) and were excluded from STREAM, and therefore from this analysis.^4^ Those without a baseline cervical smear result had a smear test after entry into the study and continued follow-up, even if a high-grade lesion was detected.

### Data source and data management

At enrolment, we recorded socio-demographic, laboratory and clinical information using a nurse-administered questionnaire and by retrospective review of clinical notes and NHLS records. We used the following validated questionnaires: Audit-C to assess alcohol use, Patient Health Questionnaire 2 (PHQ2) for depression and World Health Organization Violence Against Women for intimate partner violence. We captured data in the secure, password-protected iDataFax system (DF/Net Research, Inc., Seattle, WA, USA) and performed three rounds of data quality checks consisting of source document review, internal quality audits and weekly quality reports.

### Statistical analysis

We assessed socio-demographic and clinical exposure variables including age, educational status, having a stable partner, parity, contraceptive use, time from diagnosis to ART initiation, previous tuberculosis, intimate partner violence, alcohol use or depression and CD4 count. The primary outcome for this analysis was the absence or the presence of an abnormal cervical smear, which included atypical squamous cells of undetermined significance (ASCUS), low-grade squamous intraepithelial lesion (LSIL) and HSIL. We used descriptive statistics to summarise socio-demographic and clinical variables and missing data. Associations between socio-demographic and clinical exposures, and the outcome of an abnormal cervical smear result were assessed using bivariable and multivariable Poisson regression models with robust variance. We included covariates with a *p*-value of < 0.15 in the bivariable analysis into the multivariable model. We used SAS version 9.4 (SAS Institute Inc., Cary, NC, USA) for the statistical analysis.

## Results

A total of 390 participants were enrolled into the STREAM study, of whom 235 (60.3%) were women, with a median age of 30 years (interquartile range [IQR] 26–37 years) and a median CD4 count of 401/cells/mm^3^ (IQR 215–608 cells/mm^3^). Eight women who had HSIL at ART initiation were not eligible for enrolment and thus were excluded from the STREAM study. Of the 235 women, 176 (74.9%) women with a conclusive cervical smear result were included in this analysis (Table 1), and of the 176 women included, 66 (37.5%) had abnormal cervical smears. Amongst the women with abnormal cervical smears, 62 women (35.2%) had LSIL, three (1.7%) had HSIL and one (0.6%) had ASCUS. Women with a CD4 count < 200 cells/mm^3^ had the highest proportion of abnormal cervical smears (20/40, 50.0%). Of the 59 women with no cervical smear test result available, eight (13.6%) tests were performed but no test result was available, 13 (22.0%) were inadequate for evaluation and 38 (64.4%) had no record of testing. Median CD4 count amongst women with no smear result was 405 (IQR 244–608), compared to 401 (IQR 207–611) amongst those with a smear result (*p* = 0.498).

**TABLE 1:** Baseline characteristics of women with cervical smear results available (*N* = 176).^5^

Characteristics	Level	Overall		
		%	*n*	*N*
Age	≤ 24	21.0	37	176
	25–34	44.9	79	176
	≥ 35	34.1	60	176
Secondary education	Yes	55.4	97	175
	No	44.6	78	175
Regular or stable partner	Yes	78.4	138	176
	No	21.6	38	176
Time from diagnosis to initiation	≤ 6 months	67.1	112	167
	> 6 months	32.9	55	167
Previous history of tuberculosis	Yes	11.4	20	176
	No	88.6	156	176
Risky alcohol intake†	Yes	19.4	28	144
	No	80.6	116	144
Positive depression screen (PHQ-2 score of 2 or more)	Yes	8.5	15	176
	No	91.5	161	176
Any history of intimate partner violence	Yes	17.7	31	175
	No	82.3	144	175
CD4 count (cells/mm^3^)	≤ 500	35.2	62	176
	> 500	64.8	114	176
Children	None	19.3	34	176
	1 or more	80.7	142	176
Contraception use	Yes	14.2	25	176
	No	85.8	151	176

*Source:* Drain PK, Dorward J, Violette LR, et al. Point-of-care HIV viral load testing combined with task shifting to improve treatment outcomes (STREAM): Findings from an open-label, non-inferiority, randomised controlled trial. Lancet HIV. 2020. https://doi.org/10.1016/S2352-3018(19)30402-3. Epub ahead of print.

PHQ-2, Patient Health Questionnaire 2.

† , In women, an AUDIT-C score of 3 or more is considered positive for at-risk alcohol use.

In bivariable analysis, women with an initiation CD4 count of ≤ 500 cells/mm^3^ had a higher risk of abnormal cervical smears, compared to women with a CD4 count > 500 cells/mm^3^ (risk ratio 1.84, 95% confidence interval [CI] 1.04–3.28, Table 2). Women who had not completed secondary education also had a higher risk of abnormal cervical smears. Time from diagnosis to ART initiation, having a stable partner, previous tuberculosis, contraceptive use, intimate partner violence, alcohol use or depression were not associated with abnormal cervical smear results.

**TABLE 2:** Associations between socio-demographic variables, initiation CD4 count and abnormal cervical smear results (*n* = 176).^5^

Characteristic	Level	Abnormal cervical smear			Risk ratio		*p*	Adjusted risk ratio		*p*
		*n*	*N*	*%*	95*%*	CI		95*%*	CI	
**Initiation CD4 count (cells/mm^3^)**	> 500	15	62	24.2	1	-	-	1	-	-
	≤ 500	51	114	44.7	1.84	1.04–3.28	0.037	1.71	1.04–2.80	0.034
Age (years)	18–24	10	37	27.0	1	-	-	1	-	-
	25–34	37	79	46.8	1.72	0.86–3.47	0.126	1.52	0.77–3.00	0.233
	≥ 35	19	60	31.7	1.17	0.55–2.52	0.682	1.01	0.49–2.05	0.989
Secondary education	Yes	29	97	29.9	1	-	-	1	-	-
	No	37	78	47.4	1.58	0.97–2.57	0.066	1.48	1.02–2.14	0.038
Number of children	0	8	34	23.5	1	-	-	1	-	-
	≥ 1	58	142	40.8	1.76	0.84–3.68	0.136	1.5	0.70–3.24	0.297
Time from diagnosis to initiation > 6 months?	No	43	112	38.4	1	-	-	-	-	-
	Yes	18	55	32.7	0.86	0.50–1.49	0.594	-	-	-
Regular or stable partner	No	13	38	34.2	1	-	-	-	-	-
	Yes	53	138	38.4	1.13	0.61–2.07	0.699	-	-	-
Previous history of tuberculosis	No	56	156	35.9	1	-	-	-	-	-
	Yes	10	20	50.0	1.41	0.71–2.77	0.317	-	-	-
Contraception use	No	54	151	35.8	1	-	-	-	-	-
	Yes	12	25	48.0	1.31	0.68–2.49	0.415	-	-	-
History of intimate partner violence	No	54	144	37.5	1	-	-	-	-	-
	Yes	12	31	38.7	1.03	0.55–1.93	0.917	-	-	-
Risky alcohol intake†	No	43	116	37.1	1	-	-	-	-	-
	Yes	7	28	25.0	0.67	0.30–1.50	0.334	-	-	-
Positive depression screen (PHQ-2 score ≥ 2)	No	59	161	36.6	1	-	-	-	-	-
	Yes	7	15	46.7	1.25	0.57–2.75	0.577	-	-	-

*Source:* Drain PK, Dorward J, Violette LR, et al. Point-of-care HIV viral load testing combined with task shifting to improve treatment outcomes (STREAM): Findings from an open-label, non-inferiority, randomised controlled trial. Lancet HIV. 2020. https://doi.org/10.1016/S2352-3018(19)30402-3. Epub ahead of print.

CI, confidence interval; PHQ-2, Patient Health Questionnaire 2.

† , In women, an AUDIT-C score of 3 or more is considered positive for at-risk alcohol use.

In multivariable analyses, late initiators of ART had a higher risk of abnormal cervical smears when compared to early initiators (adjusted risk ratio [aRR] 1.71, 95% CI 1.04–2.80). Furthermore, independent of CD4 count, women with a lower educational status also had a higher risk of abnormal cervical smears (aRR 1.48, 95% Cl 1.02–2.14).

## Discussion

In this cohort of women initiating ART after implementation of UTT, there was a high prevalence of abnormal cervical lesions, affecting over a third of all women successfully screened. Women who initiated ART at lower CD4 thresholds and those who did not reach secondary education had a higher risk for abnormal cervical lesions.

Our findings are similar to a systematic review, including studies mainly from Europe and North America, which found that women with lower CD4 counts had consistently higher incidence of abnormal cervical lesions.^6^ This review also showed an increased risk of progression of cervical lesions with declining CD4 count.^6^ A cross-sectional analysis of 1140 Nigerian women showed that both low- and high-grade cervical lesions were detected almost four times more frequently in women with CD4 < 200 cells/mm^3^.^7^ Therefore, earlier initiation of ART at higher CD4 counts may have a protective effect on the development and progression of abnormal cervical lesions in this setting.^2,3,8^ Of note, in spite of the implementation of UTT, the majority of women in this study presented with CD4 counts ≤ 500 cells/mm^3^, meaning that further efforts are needed to diagnose and initiate ART earlier amongst women living with HIV.

Similar to our finding, a cross-sectional study from Zambia, analysing data in over 14 000 women from a National Cervical Screening Programme, showed that women having at least secondary education were less likely to develop abnormal cervical lesions, compared to women with no formal education.^9^ The protective effect of attaining a higher level of education may be explained by increased awareness of the disease, delayed sexual debut and greater access to healthcare, including cervical screening services. While the recent roll-out of HPV vaccination to South African school girls through a public health initiative has somewhat created renewed awareness of cervical cancer, gaps remain in knowledge and prevention of the disease and access to screening services for secondary prevention.^1,10^

Limitations of our study included the cross-sectional design and a relatively small sample size, which means that the analysis may not be sufficiently powered to rule out weak associations between other exposure variables and abnormal smears. Furthermore, we excluded women with HSIL without a conclusive negative colposcopy result from the study. Even in this large HIV clinic, a quarter of women did not have the recommended cervical smear result available at ART initiation. A strength of our study is that we were able to assess the prevalence of abnormal cervical lesions in women initiating ART at higher CD4 thresholds in the era of UTT in a public health setting.

While HPV vaccination for young women has now been introduced in South Africa, the long-term impact on cervical cancer is still uncertain. In the meantime, cervical cancer screening remains a priority. Here, we highlight that early ART initiation and ensuring frequent cervical screening for those with lower CD4 counts and lower educational background should be prioritised. Universal test and treat will help reduce cervical abnormalities, but other measures, such as scaling up of existing screening services, better colposcopy and treatment provision, as well as the implementation of high-risk HPV screening strategies, are necessary to reduce the burden of this preventable disease.
